# Projected trends in frailty prevalence and associated health service use and costs in the over-50s in England, 2025 to 2040: a simulation modelling study

**DOI:** 10.1093/ageing/afag109

**Published:** 2026-04-29

**Authors:** Bronagh Walsh, Tracey England, Sally Brailsford, Carole Fogg, Simon Fraser, Paul J Roderick, Simon de Lusignan, Scott Harris, Andrew Clegg

**Affiliations:** School of Health Sciences, Faculty of Environmental and Life Sciences, University of Southampton, Southampton SO17 1BJ, UK; School of Health Sciences, Faculty of Environmental and Life Sciences, University of Southampton, Southampton SO17 1BJ, UK; Southampton Business School, University of Southampton, Southampton, Hampshire SO17 1BJ, United Kingdom of Great Britain and Northern Ireland; School of Health Sciences, Faculty of Environmental and Life Sciences, University Road Southampton, University of Southampton, Southampton SO17 1BJ, UK; Faculty of Medicine, University of Southampton, Southampton, UK; NIHR Applied Research Collaboration Wessex; Public Health Sciences and Medical Statistics, C floor, South Academic Block, Southampton General Hospital, University of Southampton, Southampton SO166YD, UK; Nuffield Department of Primary Care Health Sciences, Primary Care Health Sciences Radcliffe Observatory Quarter Woodstock Road, University of Oxford, Oxford, Oxfordshire OX2 6GG, UK; Faculty of Medicine - Primary Care, Population Science and Medical Education, University of Southampton, Southampton, Hampshire, UK; Academic Unit for Ageing and Stroke Research, University of Leeds, UK; Bradford Institute for Health Research, Bradford Teaching Hospitals NHS Foundation Trust, Duckworth Lane, Bradford, West Yorkshire BD9 6RJ, UK

**Keywords:** frailty, service use, costs, simulation, older people

## Abstract

**Aim:**

To model projected trends in frailty prevalence, associated service use and costs in people aged 50 and over in England to 2040.

**Design:**

System dynamics simulation modelling.

**Setting:**

Adult population (aged 50 and over) of England.

**Participants:**

Routine data from primary care patients aged 50 and over (2.2 million individuals) from participating practices from the Royal College of General Practitioners Research Surveillance Centre (RCGP RSC) database between 2006 and 2017.

**Outcome measures:**

Projected frailty prevalence, primary, secondary and urgent care service use and costs in those aged 50 and over between 2025 and 2040.

**Results:**

The population of England aged 50 and over is projected to increase from 23.1 million in 2025 to 24 million in 2040. Frailty prevalence in this group will rise from 70.2% to 76.1%, with associated service use costs increasing by £10 billion. Measures to reduce frailty incidence or progression could reduce costs by £310 million/annum and £644 million/annum, respectively.

**Conclusions:**

Frailty prevalence and associated service use and costs will increase substantially in the ageing population. A shift in focus to prevention and slowing progression in middle age and the younger old would substantially reduce service use and costs by older people living with frailty.

## Key points

- Frailty prevalence in the over 50s will rise from 70.2% to 76.1% (a 5.9% increase) over the simulation period (2025 to 2040).
- Associated service use costs will increase by £10 billion by 2040.
- Measures to reduce frailty incidence could reduce costs by £310 million/annum.
- Measures to reduce frailty progression could reduce costs by £644 million/annum.

## Introduction

The UK Government aims to build a national health service in which everyone lives well for longer, requiring a focus on ensuring people enter older age in better health to maximise independence. There is similar attention internationally on maintaining health and wellbeing in ageing populations, with a shared goal to improve the lives of older people set out in the United Nations Decade of Healthy Ageing collaboration [1]. Central to this aim is the prevention and management of frailty, a condition characterised by reduced physiological reserves and resulting vulnerability to adverse outcomes, including loss of independence. Frailty is expected to become more prevalent as populations age, becoming a significant issue for health services worldwide, and identified in the 2023 Chief Medical Officer’s Report on Health in an Ageing Society as a crucial consideration for the UK health service [2]. Frailty is associated with adverse outcomes, such as high health and social care service use, unplanned admissions and transfer to residential care [3–6]. Since 2017, general practices in England have screened their populations for moderate/severe frailty, targeting patients with appropriate interventions, such as medication reviews, falls risk assessments and Comprehensive Geriatric Assessment (CGA), which have the potential to slow or prevent decline [4, 5, 7–11].

While consensus guidelines have emphasised the importance of identification and clinical management of frailty [12, 13], there is less evidence to support planning of service configuration and delivery to achieve these goals at population level, particularly in relation to the future impact of developing frailty in middle age at a population level, crucial considerations for health policy and commissioning decisions [13, 14]. Previous analyses indicate that despite lower individual costs, mild to moderate frailty in people aged up to 65 are a greater driver of costs [2, 15, 16]. The aim of this study was, therefore, to develop a simulation model of healthcare demand, service use and costs in people aged 50 and over living with frailty in England, to enable prediction of future health service impact of frailty in the ageing population and examine the impact of different health care policy priorities.

## Methods

### Study population and sample

The Frailty Dynamics (FD) computer simulation models the dynamics of frailty in the adult primary care population (aged 50 and over) in England from 2025 to 2040. Data informing model development was from 2.2 million primary care patients aged 50 and over registered in participating practices from the Royal College of General Practitioners Research and Surveillance Centre (RCGP RSC) database between 2006 and 2017 [15, 17–19]. The Secure Anonymised Information Linkage (SAIL) Databank provided a comparable cohort of 1.4 million individuals for external validation of the simulation. The simulation used Office for National Statistics (ONS) mid-year population estimates [20, 21] to estimate the number of people in each age group on 1 January 2006, aligning with the start date for routine data extraction, and ONS estimates of people turning 50, 65, 75 and 85 for ageing progression.

### Identification of frailty incidence and frailty transition rates

The electronic Frailty Index (eFI) was derived from the primary care data [3, 17]. The eFI comprises 36 deficits, including long-term health conditions, symptoms/signs, disabilities, abnormal laboratory test results and social conditions [3]. Presence or absence of a deficit was determined from the set of associated Read codes within the electronic General Practice record for each patient, with deficits totalled and divided by 36 to establish the score. Scores were categorised as fit (0 to <0.12), mild (0.12 to <0.24), moderate (0.24 to <0.36) and severe (0.36 and above), in line with the literature [3, 19, 22]. Incidence was defined as an increase in the eFI category from fit to either mild, moderate or severe frailty, and transitions were defined as an increase in the frailty category.

### System dynamics modelling

System Dynamics (SD) is a computer simulation modelling approach which is able to analyse changes over time in complex, interacting systems and is ideally suited for exploring trends in development of illness or disease in populations, as in this study [23, 24]. AnyLogic simulation modelling software (version 8.9.0) was used to build the population model, with Excel then used to derive service use and cost outputs from the population frailty projections. The SD approach allows prediction of future impact to be captured, while remaining flexible enough to explore the effect of different scenarios. These models are also easily understood by stakeholders, due to their organisational-based structure.

The SD model structure was informed by stakeholder consultation with patients, carers, commissioners, health and social care professionals [19] and the statistical analyses [15, 17–19]. A multistate model (MSM) of adjusted average annual transition rates was derived from RCGP RSC cohort data from 2006 to 2017 [18]. Starting population numbers and mortality rates were based on ONS population projections [19, 25]. The population aged 50 and over was divided into 4 age groups, reflecting groupings reported in literature relating to older adults’ healthcare and age groups of interest, including those with frailty in middle age: 50–64, 65–74, 75–84 and ≥85 [17, 19]. Each age group was further divided according to frailty severity measured by the electronic frailty index, eFI [3], resulting in 16 subgroups, referred to in the model as ‘stocks,’ with 4 frailty categories (fit, mild, moderate and severe) within each age group [25]. Ageing and mortality of the population were captured by analysis of the RCGP RSC dataset and ONS data. The individuals from the initial stocks within the 16 population subgroups were aged through progression from one age category to the next, with new individuals turning 50 also entering the model.

Yearly frailty transition rates (fit to mild, mild to moderate and moderate to severe) based on analysis of the RCGP RSC dataset and adjusted for age group, sex, ethnicity, deprivation and rural/urban location [17, 18] were applied to the four age categories. The model assumes that frailty status deteriorates or remains static over time and does not improve.

Internal and external validation against ONS, RCGP RSC and SAIL data, respectively, demonstrated that the FD Simulation Model provided robust and stable estimates of the progression of frailty in the ageing population during 2006–2017 [19, 25]. Following validation, the period identified for simulation projections was 2025 to 2040. Simulation population numbers for this period were validated against ONS population projections for those years, again demonstrating good accuracy and stability [19, 25]. The final population-level model is able to estimate future primary and secondary health care needs associated with different frailty strata in the ageing population, thus providing useful evidence for planning services for older people living with frailty.

### Simulation outputs

The underlying simulation of population ageing and frailty progression provided a robust structure on which primary and secondary care usage was overlaid. This allowed examination of service use by older people living with frailty at different ages, and exploration of the effect of different service scenarios on frailty progression and service use. The population model was used to determine numbers of new frailty cases and prevalence for future years. Service use rates from previous analysis were applied to prevalent cases to calculate the following outputs: [15] primary care use including the total number of primary care contacts (face-to-face appointments, home visits, telephone appointments and e-consultations) per year; secondary care service use including total number of outpatient visits, total number of emergency department (ED) attendances, total number of hospital admissions (also stratified by elective/unplanned admissions), total number of critical care admissions for each cohort year for each patient.

Costs were applied to service use for each year using unit costs from 2016/2017 NHS National Reference costs [26] and Personal Social Services Research Unit (PSSRU) Unit Costs of Health and Social Care data, with prices adjusted to the 2017 base [27–29] where costs were only available for other years, using an assumed inflation rate of 3.5% a year as per the analysis described elsewhere [15]. Costs from the simulation projections are provided at 2016/2017 rates for ease of comparison.

### Scenario experiments

Three policy scenarios were explored, agreed and prioritised with patient and professional stakeholders [19] and based on available evidence of potential impact of relevant interventions, as follows:

A: Reduction in frailty incidence: explored the impact of interventions to prevent onset of frailty, with the incidence rate for frailty, i.e. the rate of transitions from fit to mild frailty, reduced by 5% in all age groups [30–35].

B: Slowing of frailty progression: explored the impact of slowing progression of frailty, with a 10% reduction in mild to moderate and 5% reduction in moderate to severe transitions per year. In contrast to other scenarios, which alter only one parameter at a time, this scenario requires changes to two different transition rates applied to all age groups and results represent the combined effect of these two changes in transition rates [30, 36, 37].

C: Reduction in unplanned hospital admissions: explored the impact of interventions to reduced unplanned hospital admissions in older people with frailty, to represent the focus of clinical efforts in this area and to reflect both patient and service priorities, with a realistic 2.5% reduction in unplanned admissions per year across all frailty groups and ages [38–40].

### Patient and public involvement

In addition to patient and public involvement (PPI) representation on the study team, the study stakeholder group comprised two groups of contributors: health and social care professionals and others involved in providing or commissioning frailty services; and members of the public with either direct or indirect (e.g. carer, relative) experience of using services designed to support and manage older people living with frailty. Five stakeholder sessions were held during the study to seek input from a broad range of health and care professionals and commissioners, and five members of the general public, patients and carers [19]. These sessions informed development of the model structure, model outputs, identification of experimental scenarios and dissemination strategy.

## Results

Assuming demographic trends, frailty incidence rates and service use rates remain unchanged between 2025 and 2040, the population aged 50 and over will increase from 23 126 258 to 24 021 710 (3.9%). A total of 10 million new cases of frailty will occur during the simulation period, with the largest numbers occurring in the 50–64 year age group (Table 1).

**Table 1:** Projected incidence: number of new cases of frailty per annum (age 50 and over) in England from 2025 to 2040.

Year	50–64	65–74	75–84	85+	Overall
2025	400 200	160 300	105 400	31 100	696 900
2026	390 800	158 500	103 000	30 100	682 400
2027	380 700	158 100	100 300	28 900	667 900
2028	370 200	158 500	97 300	27 800	653 800
2029	360 200	159 100	94 300	27 400	641 000
2030	352 800	159 700	91 500	27 400	631 300
2031	348 400	160 100	88 800	27 300	624 600
2032	345 100	160 400	86 500	27 100	619 200
2033	341 900	160 100	84 200	27 900	614 100
2034	338 700	159 300	82 400	28 800	609 200
2035	335 800	158 000	81 600	28 800	604 200
2036	333 300	156 700	81 400	28 300	599 700
2037	331 500	155 100	81 900	27 600	596 000
2038	330 100	152 300	82 800	26 900	592 200
2039	329 900	148 600	83 800	26 300	588 600
2040	330 400	144 400	84 700	25 900	585 400
					
Total number of new cases of frailty	5 620 000	*2 509 209*	*1 429 900*	*447 600*	*10 006 500*

During this time, the numbers in all frailty states will increase by 12.6% from 16 228 687 in 2025 to 18 269 340 in 2040 (see Appendix 1 in the Supplementary Data Section for prevalence). Prevalence of frailty will increase from 70.2% to 76.1%, with a steep rise before levelling off towards the end of the simulation period. Over the same time period, primary, secondary and urgent care service use by people living with frailty will rise as increasing numbers in the population become frail (Table 2).

**Table 2:** Projected number of primary, secondary and urgent care use contacts in England from 2025 to 2040 (in £ millions).

	*Service use*					
*Year*	*Use of primary care services* ^a^			*Use of secondary and urgent care services* ^b^		
	*Fit*	*Frail*	*Total*	*Fit*	*Frail*	*Total*
2025	37.4	225.1	262.5	12.8	94.3	107.1
2026	36.7	229.5	266.2	12.6	96.3	108.9
2027	35.9	233.6	269.5	12.3	98.2	110.5
2028	35.2	237.4	272.6	12.1	100.0	112.1
2029	34.5	240.8	275.3	11.8	101.6	113.4
2030	33.9	243.8	277.7	11.6	103.1	114.7
2031	33.5	246.7	280.2	11.5	104.4	115.9
2032	33.2	249.4	282.6	11.4	105.7	117.1
2033	33.0	251.9	284.9	11.3	106.8	118.1
2034	32.7	254.1	286.8	11.2	107.8	119.0
2035	32.4	256.1	288.5	11.1	108.7	119.8
2036	32.2	257.9	290.1	11.0	109.5	120.5
2037	32.0	259.6	291.6	10.9	110.3	121.2
2038	31.8	261.3	293.1	10.9	111.1	122.0
2039	31.5	262.8	294.3	10.8	111.8	122.6
2040	31.3	264.2	295.5	10.7	112.5	123.2

^a^Includes General Practitioner (GP) appointments (face-to-face, telephone, home visits and e-consultations).

^b^Includes A&E attendances, critical care admissions, hospital admissions (elective and unplanned) and outpatient appointments.

The cost for providing primary care services in England in the baseline scenario will rise by £1.6 billion for people with frailty, from £8.9 billion to £10.5 billion, by 2040 (Table 3). For secondary care, the rise in costs is estimated to be £8.4 billion for patients with frailty, from £36.8 billion to £45.2 billion. The total health service cost increase will be £10 billion between 2025 and 2040.

**Table 3:** Projected service use costs (in £ millions) in England from 2025 to 2040.

*Year*	*Total primary care costs*			*Total secondary and urgent care costs*			*Total service use costs*		
	*Fit*	*Frail*	*Total*	*Fit*	*Frail*	*Total*	*Fit*	*Frail*	*Total*
2025	1430.9	8910.0	10 340.9	3607.5	36 818.6	40 426.1	5038.4	45 728.6	50 767.0
2026	1401.4	9088.4	10 489.9	3533.1	37 696.0	41 229.1	4934.6	46 784.4	51 719.0
2027	1373.0	9252.6	10 625.5	3459.9	38 508.2	41 968.1	4832.9	47 760.8	52 593.6
2028	1345.2	9403.1	10 748.3	3387.3	39 254.3	42 641.7	4732.5	48 657.4	53 390.0
2029	1318.3	9540.3	10 858.6	3319.1	39 954.0	43 273.1	4637.4	49 494.3	54 131.6
2030	1295.5	9666.2	10 961.8	3263.1	40 617.7	43 880.7	4558.6	50 283.9	54 842.5
2031	1280.9	9784.7	11 065.6	3226.2	41 248.7	44 474.9	4507.0	51 033.4	55 540.5
2032	1270.7	9895.9	11 166.6	3198.4	41 835.7	45 034.1	4469.1	51 731.7	56 200.8
2033	1260.8	9998.6	11 259.4	3172.6	42 385.9	45 558.5	4433.4	52 384.5	56 817.9
2034	1250.5	10 090.4	11 340.9	3151.1	42 924.4	46 075.5	4401.5	53 014.9	57 416.4
2035	1240.5	10 173.8	11 414.2	3127.5	43 400.0	46 527.3	4367.9	53 573.6	57 941.5
2036	1229.8	10 249.3	11 479.0	3100.4	43 822.4	46 922.8	4330.2	54 071.7	58 401.9
2037	1222.1	10 320.5	11 542.6	3080.1	44 213.8	47 293.9	4302.2	54 534.3	58 836.5
2038	1214.2	10 387.0	11 601.2	3059.5	44 576.7	47 636.2	4273.7	54 963.7	59 237.4
2039	1205.0	10 448.5	11 653.5	3036.1	44 913.9	47 950.0	4241.1	55 362.3	59 603.4
2040	1197.9	10 507.4	11 705.4	3017.6	45 238.1	48 255.6	4215.5	55 745.5	59 961.0

### Service scenario experiments

A reduction in frailty incidence of 5% (Scenario A) indicates that by 2040, the number of people with frailty would be 195 473 lower than in the baseline projections (18 073 867 compared to 18 269 340) (see Appendix 2 in the Supplementary Data Section for comparison of the scenario outputs). In this scenario, service use by older people living with frailty would decrease by ~22 million GP appointments and 11.5 million urgent and secondary care contacts, resulting in a potential cost saving of £310 million per annum, with a cumulative saving of £4.97 billion over the 16-year period.

Slowing progression of frailty through moderate and severe states (Scenario B) by 2040, the number of people with moderate and severe frailty would be 369 343 lower than in the baseline projections (9 021 522 compared with 9 390 865). Service use by older people living with frailty would decrease by ~19.5 million GP appointments and 18.8 million urgent and secondary care contacts, with a potential cost saving of £644 million per annum and a cumulative cost saving of £10.3 billion over the same period.

In contrast, a targeted approach to reduce unplanned admissions among those with frailty (Scenario C), consistent with recent clinical and policy initiatives, would only result in a reduction of secondary care use of 2.48 million unplanned admissions. By 2040, this scenario would result in a potential cumulative saving of £4.2 billion over a 16-year period.

## Discussion

This simulation provides projections of service use and costs for older people living with frailty in England over a 16-year period, adding to substantial observational evidence of a strong association between frailty and health and social care use costs [15, 41–46]. These findings demonstrate that, without action to prevent frailty onset and progression, particularly in middle and early old age, prevalence will rise by 5.9% by 2040, with associated service use costs increasing by £9.2 billion (Table 3). The projections emerging from this simulation model are in line with international literature estimates, allowing for greater confidence in the service use and cost projections [19, 47–50].

Importantly, the simulation scenario findings indicate that, while efforts to reduce service use by admission prevention in those with moderate to severe frailty will reduce costs, the impact is less than that achieved by prevention of frailty onset or progression. In reality, a combination of the above approaches is likely to be required, but in planning future services it should be remembered that, due to the relatively early onset of frailty, and the large numbers of older people living with mild to moderate frailty for long periods of time, the greatest impact on net service use and costs is likely to be achieved through slowing progression to more severe levels of frailty and prevention of frailty onset. This is unlikely to be achievable without considerable investment in public health and targeted frailty prevention interventions. Further research will be required to ascertain what interventions are most effective at achieving prevention or slowing of frailty transitions at scale in the ageing population.

Meeting the health care needs of the growing frail population will require a considerable policy shift, from the current focus on management of frailty in the oldest and most frail, to prevention in middle age or earlier. It should be noted that the prevention and progression scenarios explored in this study are conservative, but realistic given that adherence to such interventions is generally low and more work is needed on translation to community settings [51, 52]. Greater changes could be possible with targeting of resources; cohort studies have demonstrated reductions in frailty incidence of up to 20% with lifestyle changes, such as physical activity, diet and smoking cessation [30, 32, 34, 35, 53]. There is also a need for future research on engaging the public with such interventions, and exploration of the workforce requirements and cost-effectiveness of such an approach.

### Policy and practice implications

The findings of this simulation study are globally important, with implications for all health services serving ageing populations. Service providers need to consider that frailty is likely to already be present in the population before age 65, and the average age of onset of frailty may be lower than expected [2, 17, 18]. In addition, even where frailty incidence rates are stable, a continuation of current trends in frailty onset and progression will result in steeply rising prevalence of frailty as populations age, with associated increases in service use and costs. These analyses indicate that health and social care services will need to take into account that appropriately tailored and evidenced interventions to manage patients to delay further progression will be required, as well as properly resourced health and care systems to cope with the increased service use expected in ageing populations. In addition, delaying onset of frailty will be important in reducing downstream service use and costs associated with frailty, particularly in middle age. Targeted prevention at national population level has the potential to result in substantial cost savings, but requires development and evaluation of services specific to older people at risk of frailty and frailty progression. Service planners should also note that scenario modelling in this study revealed that reductions in service use and costs achieved through reduced frailty incidence and progression are, to some extent, offset by continued service use and costs in less frail states.

### Limitations

In simulation modelling, findings are constrained by the model assumptions, structure and parameters selected. In this case, the underlying assumption was that current incidence rates, progression, demographic trends and service provision and costs would remain stable over the baseline simulation period. Model development was informed by analysis of cohort data from 2006 to 2017, with average annual frailty transitions from this cohort applied in the model for projections of future impact. A fundamental assumption of the model is that incidence and progression rates will remain stable [18], but these rates could be revised if evidence emerges of changes to the underlying rates. Professional and public members of the stakeholder engagement group also agreed that the impact of deprivation and the COVID-19 pandemic on frailty prevalence will be important avenues for future research when there is sufficient evidence to inform relevant model parameters. While it is unlikely that health services will remain unchanged over the simulation period, this approach allowed exploration of the independent impact of population ageing on service use associated with older people living with frailty, the first simulation study to explore this issue.

As with all simulation models, simplifications have been made in the development of the FD model. Reversals in frailty status were not included in the simulation due to their small numbers and uncertainty about the identification of such events using eFI [19]. However, reversal in frailty state could become a key goal of future frailty services. Revisions to the eFI tool will allow measurement of reversals in frailty state [49]; incorporation of data on such reversals into the simulation would therefore greatly increase its utility in modelling the impact of future services.

In relation to model parameters, a strength of this simulation study is its use of large-scale routine health care data with a long period of follow-up. GP registration coverage in England is high [54]; therefore, using data from the primary care population ensured that findings are representative of the overall population. The simulation model has undergone extensive internal and external validation against real-world data and has been shown to be both robust and accurate [19, 55]. It should be noted, however, that incidence in the youngest age group had a marked effect on the overall prevalence, because age groups are unequal in size and numbers in that group are so large compared with other age subgroups. Age groups could have been reconfigured into a larger number of similarly sized groups. The study stakeholder group agreed, however, that retaining the 50–64 age group was important in terms of clinical practice and service organisation for frailty in middle age, a key focus of the study. These findings emphasise the potential long-term impact of frailty within this age group and support the argument for screening and prevention as early as possible.

Scenario assumptions were conservative, reflecting literature estimates [30–35, 39, 40, 51, 56], and consultation with professionals [19, 31, 37], which cautioned that while improvements in frailty progression and admissions are possible with a range of interventions, any improvements in practice are likely to be modest. This ensured that proposed changes are deliverable at system level, but potential benefits could have been under-estimated.

Finally, the simulation only considered publicly funded health care costs and not total care costs associated with frailty. Collection of data on public sector social care and community-based health care costs were beyond the scope of this study; a gap being addressed in ongoing work [57]. The costs of privately funded and unpaid informal care could not be addressed through available, linked routine data. There is, however, evidence that private home-based care comprises ~25% of home care costs [58–60], providing an indication of the likely scale of additional formal care costs for this population.

## Conclusion

This study has modelled the future impact of frailty in the ageing population of England, showing that frailty prevalence will increase substantially by 2040. Associated service use and costs will also rise without prevention or slowing of frailty progression, particularly in the large numbers of people at risk of frailty, or living with mild frailty, in middle age. While these interventions would have substantial impact on service use and costs, they would require a major shift in policy and practice around older people living with frailty.

## Supplementary Material

aa-25-2377-File002_afag109

aa-25-2377-File003_afag109

## Research Data Transparency and Availability:

The data used in the study are secondary data and are retained by the data providers (RCGP RSC and SAIL).
