# Supplementary figures and images for: Projected trends in frailty prevalence and associated health service use and costs in the over-50s in England, 2025 to 2040: a simulation modelling study
Source: Age Ageing. 2026 Apr 29;55(4):afag109. doi: 10.1093/ageing/afag109 (PMC13127134; doi:10.1093/ageing/afag109)

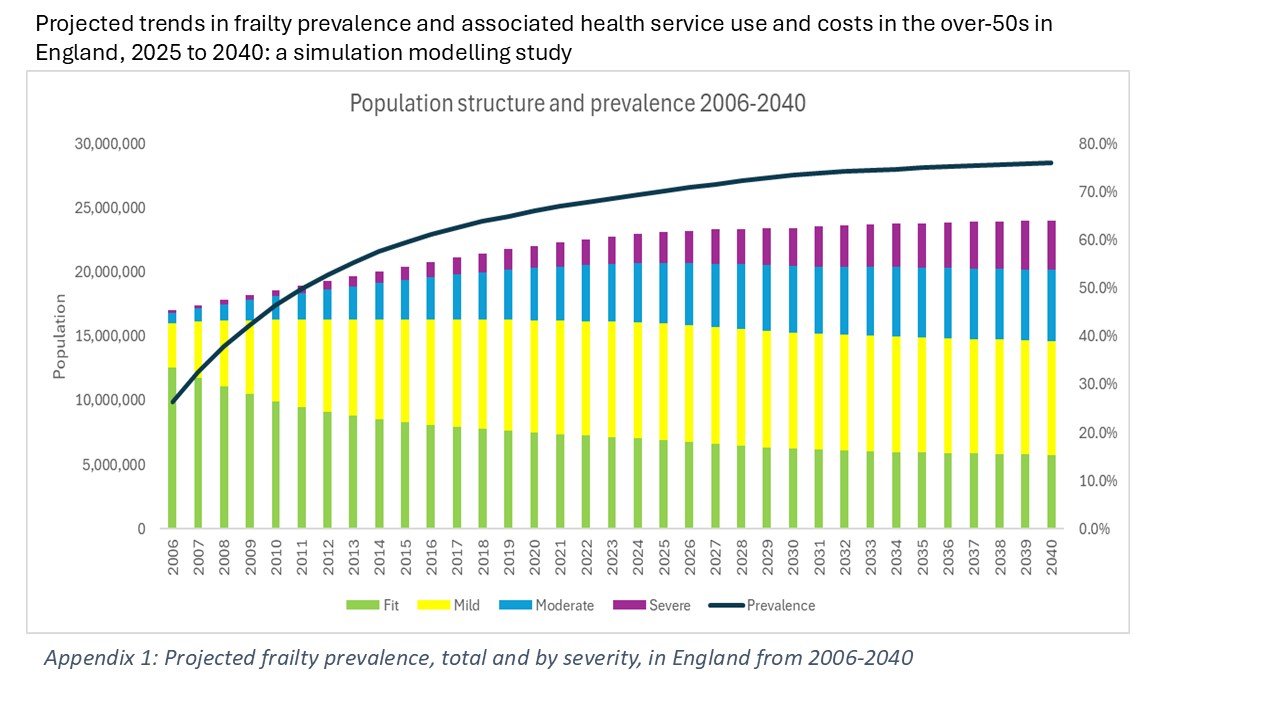

Supplement: aa-25-2377-File002_afag109 [file aa-25-2377-file002_afag109.jpeg]

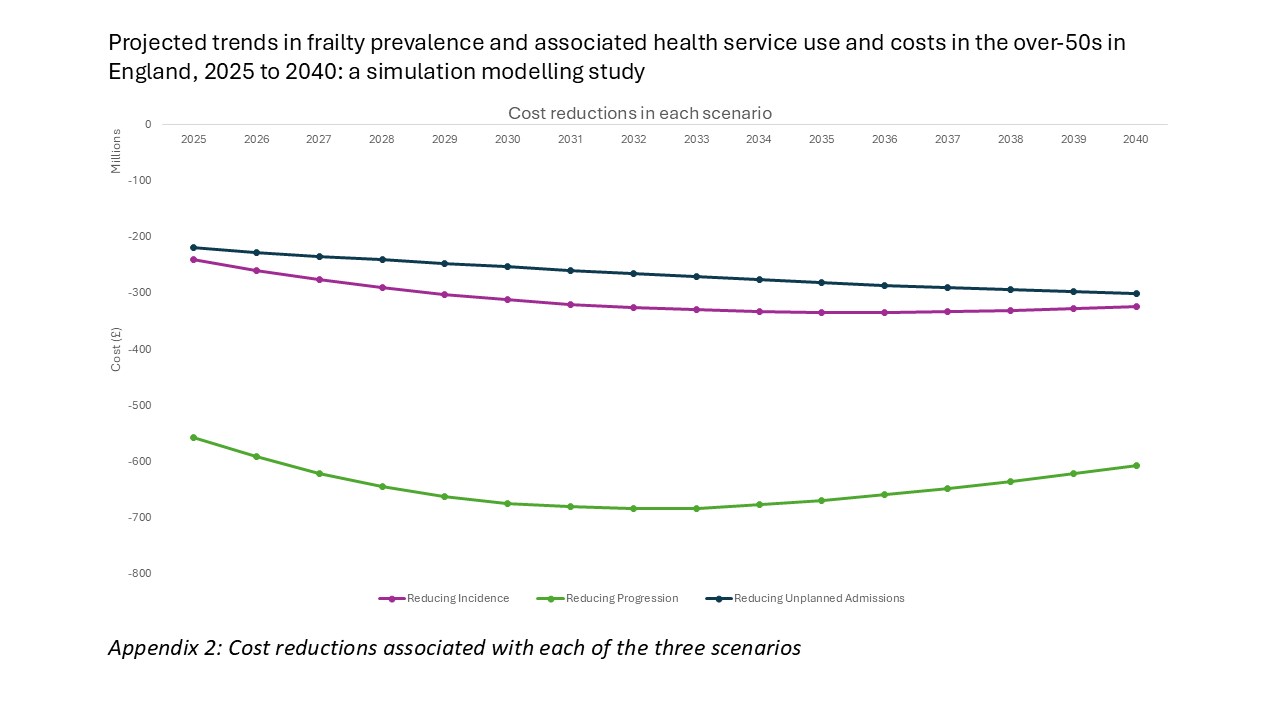

Supplement: aa-25-2377-File003_afag109 [file aa-25-2377-file003_afag109.jpeg]
